# Prevalence and risk factors for post-investigation colorectal cancer (“interval cancer”) after computed tomographic colonography: protocol for a systematic review

**DOI:** 10.1186/s13643-017-0432-8

**Published:** 2017-02-21

**Authors:** Andrew A. Plumb, Anu Obaro, Thomas Fanshawe, Ulysses S. Torres, Rachel Baldwin-Cleland, Steve Halligan, David Burling

**Affiliations:** 1 Centre for Medical Imaging, University College London, 3rd Floor East, 250 Euston Rd, London, NW NW1 2PG UK; 2 St. Mark’s Academic Institute, St. Mark’s Hospital, Harrow, UK; 3 Nuffield Department of Primary Care Health Sciences, Oxford University, Oxford, UK; 4 Department of Radiology, Federal University of Sao Paulo, Sao Paulo, Brazil

**Keywords:** Colorectal neoplasms, Computed tomographic colonography, Systematic review, Meta-analysis, Protocol

## Abstract

**Background:**

Colorectal cancer (CRC) is a common and important disease. There are different tests for diagnosis, one of which is computed tomographic colonography (CTC). No test is perfect, and patients with normal CTC may subsequently develop CRC (either because it was overlooked originally, or because it has developed in the interim). This is termed post-investigation colorectal cancer (PICRC) or “interval cancer”. How frequently this occurs after CTC is not known. The purpose of this systematic review and meta-analysis is to use the primary literature to estimate the PICRC rate after CTC, and explore associated factors.

**Methods:**

Primary studies reporting post-investigation colorectal cancer (PICRC) rates after CTC will be identified from PubMed, Embase and Cochrane Register of Controlled Trials databases. Peer-reviewed studies published after 1994 (the year CTC was introduced) will be included and the rate of PICRC within 36 months of CTC recorded. Data will be extracted from selected studies for a random effects meta-analysis. Heterogeneity, risk of bias and publication bias will be assessed, and exploratory analysis will examine factors associated with higher PICRC rates in the literature.

**Conclusion:**

PICRC rates are the ultimate benchmark of diagnostic quality for colonic investigations. This systematic review and meta-analysis will identify and synthesise evidence to determine PICRC rates after CTC and explore factors that may contribute to higher rates.

**Systematic review registration:**

PROSPERO (registration number CRD42016042437).

**Electronic supplementary material:**

The online version of this article (doi:10.1186/s13643-017-0432-8) contains supplementary material, which is available to authorized users.

## Background

### Rationale

Colorectal cancer (CRC) is a common disease with high morbidity, mortality and healthcare costs, affecting over half a million people each year globally [1], ultimately proving fatal in approximately 50% of cases. Early disease has an excellent prognosis, whereas diagnosis at a late stage carries much higher mortality [2], indicating the importance of having rapid, easily accessible and highly sensitive diagnostic tests that are acceptable to patients. Diagnosis of CRC at an early, readily-treatable stage (for example, by population screening) has been proven by meta-analysis of several randomised trials to reduce disease-specific mortality [3]. However, this is not the only benefit of CRC screening. The prevailing hypothesis of colorectal carcinogenesis postulates that most CRC develop from potentially premalignant precursor polyps; either adenomas (the so-called adenoma-carcinoma hypothesis [4]) or serrated lesions (the “serrated pathway” [5]). In support, removal of adenomas via endoscopic polypectomy reduces the incidence of subsequent CRC [6], proving beyond reasonable doubt that a proportion of adenomas are destined to transform into malignant CRC. Therefore, timely investigation of the colon improves CRC outcomes in two broad ways—firstly, early detection of established CRC improves disease-specific mortality [3]; and secondly, identification and removal of CRC precursors reduces disease incidence [6].

The most common tests for investigation of the whole colon (as opposed to just the more distal portion) are optical colonoscopy (OC) and computed tomographic colonography (CTC). OC uses a small video camera mounted on a flexible tube that can be navigated around the entire colon to identify abnormalities, including CRC and polyps [7]. Furthermore, OC permits simultaneous polyp removal (polypectomy) using instruments that can be delivered down the colonoscope channel. CTC relies on rapid, high-resolution CT scanning of the prepared, gas-distended colon. Polyps and cancers are depicted by their disruption of the normal smooth wall of the colon or by ancillary features such as wall thickening [8].

The diagnostic accuracy of CTC has been studied extensively, with meta-analysis suggesting that diagnostic sensitivity for established CRC is equal to that of OC [9]. However, CTC is less sensitive than OC for small (6–9 mm) and diminutive (0–5 mm) polyps, with one meta-analysis suggesting approximately 70% sensitivity for 6–9 mm lesions [10]. In the majority of cases, this is of no clinical consequence because the large majority of small and diminutive polyps rarely harbour high-risk premalignant features or invasive CRC [11]. However, the precise risk is unknown for any given individual. Therefore, it is possible that the rates of post-investigation CRC (PICRC) after CTC are greater than after colonoscopy because of the small but non-zero risk of a small or diminutive polyp transitioning to invasive CRC. Although PICRC rates after colonoscopy have been reported widely [12–16], and been the subject of systematic review [17], this is not true for CTC. This hampers physicians’ and policy-makers’ ability to provide evidence-based recommendations regarding suitable follow-up intervals after a negative CTC examination.

Here, we report the protocol for a systematic review (SR) and meta-analysis of PICRC rates after negative CTC.

### Objectives

The present study will systematically review and, if possible, meta-analyse the available literature reporting PICRC rate following CTC. We wish to estimate the prevalence of PICRC in patients undergoing CTC, the uncertainty surrounding this estimate and explore potential explanatory factors associated with higher PICRC rates. Since PICRC may occur at variable times after an index text, we will examine several time horizons, ranging from 1 to 5 years after CTC. We will also assess and document the quality of the available literature, and, if necessary, make recommendations for future research and study reporting. This protocol is registered with PROSPERO (CRD42016042437) and has been reported in accordance with the PRISMA-P guidelines (see Additional file 1—PRISMA-P checklist) [18].

## Methods

### Eligibility criteria

Studies will be selected according to the criteria below.

#### Study design and participants

We will include randomised controlled trials (RCTs), cohort, cross-sectional and case-control studies reporting original data for in vivo research in adult human participants, and reporting a PICRC rate (or sufficient data for such a rate to be calculated, see definitions below). Studies will be grouped together to calculate the PICRC rather than treating them separately. We will restrict our search to peer-reviewed, full-research reports. Studies in which the CTC examination was conducted immediately following colonoscopic diagnosis of CRC (e.g. to complete colonic imaging upstream of a stenosing colonic tumour, “completion CTC”) will be excluded, unless these patients constituted less than 50% of the imaged population or were presented as a separately identifiable subset that can be excluded during data extraction. We will examine the effect of including studies with such high rates of completion CTC on the estimated PICRC rate by conducting a sensitivity analysis, in which we will re-calculate this rate after excluding studies with prevalences of completion CTC from 0 to 50% in 10% increments.

#### CTC test methods

We will define CTC as CT scanning of the prepared, gas-distended colon. Only studies in which CTC was conducted with at least dual patient positioning, gas distension and either bowel cleansing or faecal tagging will be included. We will not stipulate a particular mode of interpretation of CTC (e.g. two-dimensional vs three-dimensional) since both are used in clinical practice.

#### Definition and identification of CTC-detected cancers and PICRC

We will define a CTC-detected cancer as having occurred if either (1) direct inspection of radiology reports permitted the study authors to determine that CTC diagnosed a CRC or (2) CRC was diagnosed within 6 months of CTC occurring, and the CTC report was not available for review (in common with the OC literature, in which CRC diagnosed within 6 months of an index OC are assumed to have been detected by that test [12–16]), whereas diagnoses after that period are assumed to be PICRC. We will record which of these possibilities applies to each included study and report outcomes for these two groups of studies separately. We anticipate that studies from group (1) will be RCTs or smaller observational series, in which CTC reports are freely available to investigators, and studies from group (2) will be larger-scale population-based studies using database linkage methods in which full CTC reports may not be available.

A PICRC will be defined as diagnosis of CRC within 5 years of CTC that did not detect a cancer (i.e. did not meet either criterion 1 or 2 above). Recurrent CRC after a positive initial CTC will therefore not be regarded as PICRC. We will require studies to report a minimum period of 12 months follow-up after CTC, since shorter follow-up periods are likely to miss a substantial proportion of PICRC (which may not reach medical attention until beyond this time period). PICRC must be identified by reference to a cancer registry or intelligence network that is at least regional in scope (i.e. encompassing multiple hospitals from beyond a single municipal area), or with each individual’s true disease status determined at follow-up by a dedicated whole-colon examination (either colonoscopy, repeat CTC or colon capsule). If patients do not undergo a definitive follow-up examination, identification of PICRC solely by searching local cancer databases, registries or multidisciplinary team meeting records will not be acceptable, as PICRC could be detected at institutions remote from those conducting the index CTC. We will record the nature and scope of the registry by which PICRC cases are identified for each individual research study.

#### Setting and language

There will be no restriction by study setting. We will include reports in English, French, Spanish and German, arranging for translation where necessary. We will provide a list of potentially eligible articles from other languages in an appendix.

### Information sources

We will use the Ovid SP interface to search MEDLINE and Embase, and the Wiley interface to search the Cochrane Register of Controlled Trials, from 1994 onwards (the year that CTC was first described [19, 20]). We will also scan the reference list of included studies and any relevant narrative reviews identified by the search. Finally, we will compare the list generated by the search with studies already known to the investigators.

### Search strategy

The search strategy has been devised in conjunction between the study investigators, an information scientist and other experts in the field of CTC and imaging research. We will use a combination of medical subject headings (MeSH) and free-text terms relating to CTC and colorectal cancer. For CTC, the MeSH terms will be: “Colonography, Computed Tomographic”, which has replaced the previous terms “CT Colonography”; “Colonography, CT”; “Colonoscopy, Virtual”; “Computed Tomographic Colonography”; and “Virtual Colonoscopy”. A preliminary search has revealed no incremental benefit from including the older MeSH terms. The free-text terms will be ((CT or (comput* and tomogra*) and colonogra*) or (virtua* and colonosc*)). These will be combined using the “OR” operator, to return articles relating to CTC. Subsequently, we will search for articles regarding colorectal cancer by using the MeSH term “Colorectal Neoplasms”, combined with the free-text terms “colorectal” and “cancer” using the “OR” operator. Finally, we will combine the two searches (i.e. colorectal cancer and CTC) as below:

1. ((CT or (comput* and tomogra*)) and colonogra*).af
2. (virtua* and colono*).af
3. 1 or 2
4. colonography, computed tomographic.sh
5. 3 or 4
6. 5 and Journal Article.pt
7. ((colon or colorect*) and (cancer or carcinoma)).af
8. colorectal neoplasms.sh
9. 7 or 8
10. 9 and Journal Article.pt
11. 6 and 10

We will use the Ovid SP interface “Filters” options to restrict to articles reporting human research published after 1994.

### Selection process

Two independent review authors will screen all titles and abstracts of the articles identified by the search described above against the eligibility criteria. We will obtain the full text article for all reports that then appear to meet our criteria, or where there is uncertainty. If there is persistent uncertainty after reviewing the full text reports, we will attempt to contact the corresponding author of the study in question. Disagreement will be resolved by discussion between co-authors, and we will record the reasons for excluding each individual study identified by the search string. The number of articles included and excluded at each stage of the selection process will be summarised using a flowchart. We will report the level of inter-rater agreement between the authors performing such selection.

### Data management and extraction process

The results of the search above will be retrieved to an Endnote ×7 (Thomson Reuters, Toronto, ON, Canada) database that will be shared between the reviewers using the online interface. Subsequently, each reviewer will export potentially eligible abstracts to a new Endnote library. These will be combined and full text articles retrieved, thereby facilitating subsequent full text article screening. We will use the de-duplication functions of Ovid SP at the search stage and of Endnote ×7 at the article screening stage.

Once the final set of eligible articles has been identified, we will extract relevant data (detailed below) from each article into a Microsoft Excel 2016 spreadsheet designed specifically for the review (see Additional file 2—Extraction sheet). All articles will be extracted by two authors working independently and after extracting data from 10 articles, the agreement between the two authors will assessed. If agreement is lower than 80%, we will retain dual independent extraction. All differences will be resolved by discussion between the disagreeing parties, with a third senior author arbitrating where necessary.

We will contact corresponding authors for articles in which data items necessary to inform the primary outcome are not available from the full text reports. Duplicate reports of the same patient data set (i.e. duplicate, overlapping or companion studies) will be combined and extracted as a single study. If there are logical inconsistencies between these duplicate reports, or if overlapping data cannot be separated into extractable constituents, we will contact the corresponding author(s) of the article in question for clarification and exclude the article if no response is received within 30 days.

### Data items

We will extract the following: (a) study characteristics—primary author, year of publication, period of patient recruitment/inclusion, geographical location of the population studied, study design and number of centres involved, length of follow-up to permit post-CTC cancers to be identified; (b) patient characteristics—number of patients included, reason for undergoing CTC, gender distribution and age distribution; (c) CTC test characteristics—number of CTC examinations conducted, number completed successfully, number analysed, mode of bowel preparation, use of faecal tagging, CT scanner type (multi- vs single-row detector row), exposure factors of kVp and mAs, reconstruction interval and use of intravenous contrast; (d) radiologist/reader characteristics—predominant mode of interpretation (i.e. primary two-dimensional, primary three-dimensional or mixed) and reader training and experience; (e) tumour characteristics—number of patients with CRC detected by CTC (see definition above for detected CRC), number of patients with CRC not detected by CTC (i.e. PICRC, see definition above), location (proximal vs distal) of detected and missed cancers, temporal interval between the index CTC and the diagnosis of PICRC, and whether or not the PICRC was visible in retrospect on the CTC images.

### Outcomes

The primary outcome will be the prevalence of PICRC at 36 months follow-up after CTC, since this is the time horizon most commonly reported in the OC literature [12–16]. This will be expressed as the proportion of PICRC from the total of cancers detected (i.e. using the number of CRC as the denominator) and, secondarily, as the proportion of CTC examinations leading to a PICRC (i.e. using number of CTC examinations as the denominator). The latter figure is subject to variation as a result of lesion prevalence but may be more representative of what may be expected in routine clinical practice. By analogy with sensitivity for a diagnostic test, the total number of CRC will include those detected by both the index test and by subsequent follow-up (i.e. “disease positive”). If studies do not report data for 36 months follow-up, we will nonetheless extract the date for the closest timepoint to 36 months that is reported. Secondary outcomes will be the prevalence of PICRC at 5 years (60 months) follow-up (i.e. the current recommended screening interval for CTC [21]); incidence of PICRC per 1000 person-years of follow-up; the anatomical distribution (i.e. proximal vs distal colon) of detected CRC and PICRC; CTC scan technique, radiologist and patient characteristics associated with higher rates of PICRC; the proportion of PICRC that were visible on the images in retrospect (and potential reasons for these being missed initially) and literature quality (see subsequent section).

### Quality assessment and risk of bias of individual studies

We will assess the quality of included studies using a pre-specified quality assessment tool (), adapted from the Newcastle-Ottawa Scale (NOS) for non-randomised studies (see Additional file 3—Modified NOS) [22]. We do not anticipate assigning studies an overall quality score by pooling individual elements, as this may be unreliable [18]. We will report the overall components of the quality assessment for each study graphically using the “star rating” system of the NOS. Studies with no stars for any of the individual components of the NOS (selection, comparability and outcome assessment) will not be included in the quantitative synthesis.

### Data synthesis

If the studies are sufficiently homogenous in their design, outcome assessment and follow-up, we will conduct meta-analyses using a random effects model (DerSimonian and Laird [23]) using the current version of R (R Foundation for statistical computing, Vienna, Austria) [24]. We will use the relatively more conservative random effects model as we expect studies to have included a variety of patient participants (e.g. screening and symptomatic populations) and use radiologists of varying expertise. We will combine the percentage of patients with PICRC in each individual study to estimate a pooled prevalence, along with a 95% confidence interval (CI). If studies report variable lengths of follow-up preventing meta-analysis of prevalence at a 36-month time point, we will attempt to estimate a survival curve for PICRC from the individual study estimates using random effects survival meta-analysis [25]. If available, we will compare the prevalence of proximal and distal PICRC using an odds ratio (OR) with 95%CI. We will assess heterogeneity between study-specific estimates using the inconsistency index (*I* ^2^ statistic [26]). If heterogeneity is considerable (*I* ^2^ > 75%) and the *p* value <0.1, we will not perform quantitative data synthesis [18]. We will investigate between-study sources of heterogeneity using subgroup analyses by stratifying original estimates according to study characteristics, CTC technique and radiologist factors as described in the data extraction section above. We plan to investigate for small study effects (and possible publication bias) both qualitatively, by inspecting funnel plots [27], and quantitatively, using the test proposed by Harbord [28], although we will defer a decision regarding the suitability of these methods until the number of included studies and between-study heterogeneity is known. If there is evidence of possible publication bias and heterogeneity is sufficiently low (*I* ^2^ < 25%) [26], we will estimate PICRC rates using the trim and fill method [29] to gain an approximation of the “missing” study rates and overall summary estimate and will present this as an estimate of the potential impact of missing studies.

### Confidence in cumulative estimate

The strength of the overall weight of evidence for the primary outcome will be judged using the Grading of Recommendations Assessment, Development and Evaluation (GRADE) working group methodology [30]. This will encompass the key domains of risk of bias, consistency, directness, precision and publication bias, and will incorporate the quality assessments of individual studies and estimates of publication bias described above (see above). We will consider all articles that are selected for the systematic review, even if they are excluded from the quantitative meta-analysis. The overall research literature quality will be summarised as high (we are very confident that the true PICRC rate lies close to the estimate presented), moderate (we are moderately confident in the PICRC rate presented), low (we have limited confidence in the estimated PICRC rate) or very low (we have very little confidence in the calculated PICRC rate, i.e. the true PICRC rate is likely to be substantially different), in accordance with GRADE recommendations.

## Discussion

CTC is a widely available and commonly-used diagnostic test, with over 80,000 examinations performed each year in England alone [31] (predicted to rise to 150,000 per year by 2020 [32]). Although diagnostic accuracy has been estimated by prior meta-analysis [9, 10, 33–35], the longer-term effect of the test’s relatively lower sensitivity for small colorectal polyps (vs. the main competing technology, colonoscopy) is not known. It is plausible that this translates to higher rates of PICRC than colonoscopy. Conversely, the opposite is also possible—for example, if incomplete colonic evaluation was commoner at colonoscopy than CTC, then this might translate to higher PICRC rates. The technical failure rate of CTC is extremely low in routine clinical practice (at around 0.7–2.0% [36, 37]), meaning this scenario is at least possible. For example, in a randomised study comparing CTC and colonoscopy in symptomatic patients, the rate of clinical uncertainty/inadequate examinations after CTC was half that of colonoscopy [38]. This systematic review will permit an estimate of the prevalence of PICRC and explore factors that may contribute to missed lesions. Predetermined measures of study quality and publication bias will be used to describe the confidence in the published literature. Possible limitations to the execution of this systematic review include limited primary literature with incomplete reporting, precluding ability to extract the necessary data. However, we anticipate that the review will be of value in planning quality markers for CTC and in designing rationalised follow-up and repeat imaging schedules for patients with a negative initial CTC examination.

## Additional files

Additional file 1: PRISMA-P 2015 checklist. Description: Completed PRISMA-P checklist document. (DOCX 32 kb)
Additional file 2: Extraction Sheet for PICRC systematic review. Description: Extraction sheet to be used by two independent authors to collate data from eligible studies. (XLSX 10 kb)
Additional file 3: Newcastle-Ottawa Scale modified for CT Colonography. Description: Modified Newcastle-Ottawa Scale for CT colonography to assess quality of studies eligible for this systematic review. (XLSX 11 kb)

## Abbreviations

CI: Confidence interval
CRC: Colorectal cancer
CT: Computed tomography
CTC: Computed tomographic Colonography
GRADE: Grading of Recommendations Assessment, Development and Evaluation
*I*^2^: Inconsistency Index
MeSH: Medical subject headings
NOS: Newcastle-Ottawa Scale
OC: Optical colonoscopy
OR: Odds ratio
p: Probability value
PICRC: Post-investigation colorectal cancer
PRISMA: Preferred Reporting Items for Systematic Reviews and Meta-Analyses
PROSPERO: Prospective Register of Systematic Reviews
RCT: Randomised controlled trial
SR: Systematic review
